# Predicted osteotomy planes are accurate when using patient-specific instrumentation for total knee arthroplasty in cadavers: a descriptive analysis

**DOI:** 10.1007/s00167-017-4721-5

**Published:** 2017-09-25

**Authors:** A. J. Kievit, J. G. G. Dobbe, G. J. Streekstra, L. Blankevoort, M. U. Schafroth

**Affiliations:** 1 Orthopaedic Research Center Amsterdam, Department of Orthopaedic Surgery of the Academic Medical Center, University of Amsterdam, Amsterdam Movement Sciences, 1100 DE Amsterdam, The Netherlands; 2 Department of Orthopedics of the Tergooi hospital, Hilversum, The Netherlands; 3 Department of Biomedical Engineering and Physics of the Academic Medical Center, University of Amsterdam, 1100 DE Amsterdam, The Netherlands

**Keywords:** Total knee arthroplasty, Biomet *Signature*, patient-specific instrumentation, Accuracy study, 3D Analysis, CT

## Abstract

**Purpose:**

Malalignment of implants is a major source of failure during total knee arthroplasty. To achieve more accurate 3D planning and execution of the osteotomy cuts during surgery, the *Signature* (Biomet, Warsaw) patient-specific instrumentation (PSI) was used to produce pin guides for the positioning of the osteotomy blocks by means of computer-aided manufacture based on CT scan images. The research question of this study is: what is the transfer accuracy of osteotomy planes predicted by the *Signature* PSI system for preoperative 3D planning and intraoperative block-guided pin placement to perform total knee arthroplasty procedures?

**Methods:**

The transfer accuracy achieved by using the *Signature* PSI system was evaluated by comparing the osteotomy planes predicted preoperatively with the osteotomy planes seen intraoperatively in human cadaveric legs. Outcomes were measured in terms of translational and rotational errors (varus, valgus, flexion, extension and axial rotation) for both tibia and femur osteotomies.

**Results:**

Average translational errors between the osteotomy planes predicted using the *Signature* system and the actual osteotomy planes achieved was 0.8 mm (± 0.5 mm) for the tibia and 0.7 mm (± 4.0 mm) for the femur. Average rotational errors in relation to predicted and achieved osteotomy planes were 0.1° (± 1.2°) of varus and 0.4° (± 1.7°) of anterior slope (extension) for the tibia, and 2.8° (± 2.0°) of varus and 0.9° (± 2.7°) of flexion and 1.4° (± 2.2°) of external rotation for the femur.

**Conclusion:**

The similarity between osteotomy planes predicted using the *Signature* system and osteotomy planes actually achieved was excellent for the tibia although some discrepancies were seen for the femur. The use of 3D system techniques in TKA surgery can provide accurate intraoperative guidance, especially for patients with deformed bone, tailored to individual patients and ensure better placement of the implant.

## Introduction

Malalignment or an incorrectly sized implant is the major cause of failure in total knee arthroplasty (TKA) [12]. In conventional TKA preoperative planning, patients are assessed on the basis of standing anteroposterior and lateral radiographs, sunrise view of the patella or standing whole-leg radiographs to determine the mechanical and anatomical axis. The aim of preoperative planning and assessment of the tibia and femur is to determine the quality of bone stock, to estimate correct relative axial rotational and translational alignment and the position of the joint line and also to select a correctly sized implant. The additional benefits of accurate planning are shorter operation times and reduced risk of complications.

Until recently, preoperative planning based on 2D radiographs was the recommended method to prepare for total knee arthroplasty (TKA). However, recent studies have shown that 2D preoperative methods are not always reliable for TKA [1–3, 14, 16]. More accurate 3D computer-assisted techniques are now being employed; for example, the navigation techniques in computer-assisted surgery (CAS) help increase alignment accuracy [5, 9].

Likewise, 3D patient-specific instrumentation (PSI) systems are increasingly used in preoperative planning for TKA to predict the alignment of osteotomy planes. Currently, nine commercial PSI systems are available for use in knee arthroplasty procedures [19] of which the *Signature*™ Personalized Patient Care system (Biomet *Signature* Knee System: in collaborative partnership with *Materialise* NV) is most commonly used [25]. The *Signature* technique processes data from preoperative CTs or MRIs of patients’ entire lower limbs to produce patient-specific guides that match each individual’s anatomical geometry. These patient-specific guides ensure optimal placement of the stainless steel mechanism guiding the oscillating saw that cuts the planes in tibia and femur. The aim of patient-specific guides is to improve the accuracy between predicted and achieved osteotomy planes and thus reduce operation time and the risk of complications. Moreover, this technique does not cause intramedullary damage, in theory, reducing the risk of fat embolisms [13] although this claim has not yet been proven. A further advantage of such a system is for use in patients where standard anatomical landmarks are unreliable because of bone deformation caused by (iatrogenic) trauma or developmental problems.

The added value of PSI has been questioned in recent studies [4, 6, 18, 23], even though more than 80,000 PSI-assisted operations were performed in 2012 worldwide [25]. Many of these recent PSI studies only looked at the final position of the implant as a measure of success. However, implant position does not necessarily indicate that the optimal osteotomy plane was actually achieved as the cement used can obscure the planes. It is clear that to justify the use of PSI, the prediction and orientation of achieved osteotomy planes should be better, or at least as accurate, as conventional 2D systems reported in the literature.

Therefore, this study aims to show the added value of a 3D based system for predicting the position and orientation of osteotomy planes preoperatively in individual patients so as to provide accurate intraoperative guidance and ensure better placement of the implant and a greater chance of recovery.

## Materials and methods

### 3D prediction, planning and surgery

The preoperative prediction study was performed using CT images of nine fresh-frozen whole human legs (foot to femur head) as data for the 3D *Signature* Personalized Patient Care software (Biomet, Warsaw, USA). The specimens had a median age of 82 years (min–max 71–92; six males and three females; six right and three left limbs). The CT was chosen for scanning, as images are considered more accurate than those from MRI [26]. CT scans were made using a Brilliance 64-channel CT scanner (Philips Healthcare, Best, the Netherlands). A clinical scanning protocol was used to make the CT scans for both the planning and evaluation of the actual cutting planes. The osteotomies of interest were the distal femur cut, the posterior femur cut and the proximal tibia cut as they are made using pin placement via the guides. The femur osteotomy planes were predicted using the *Signature* software with zero degrees of extra varus/valgus (coronal projection) adjustment along the anatomical axis, three degrees of flexion (sagittal projection) in the femur and zero degrees of rotation in the axial projection. The tibia osteotomy was predicted at a standard zero degree of varus/valgus and zero degrees of posterior slope. Subsequently, the researchers sent the CT data sets via *Biomet* to *Materialise* (Leuven, Belgium), who manufactured the specific femur and tibia guides and sent these to the surgeon.

**Fig. 1:**
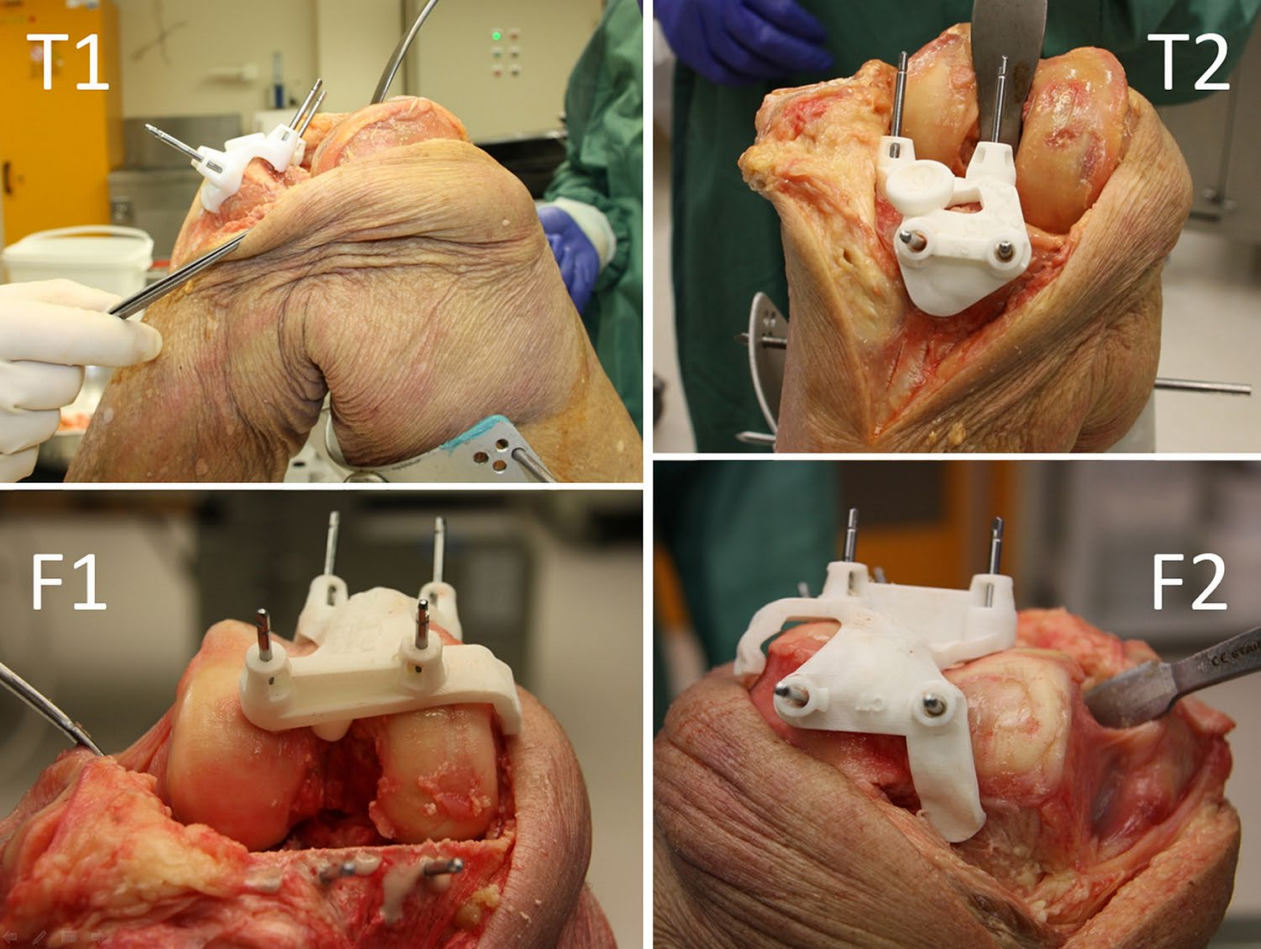
Pin-positioning guides snugly fit to the proximal tibia in lateral view (T1) and anteroposterior view (T2). The femoral guide is shown from distal to proximal, above being the anterior femur (F1), and from anterior to posterior, above being the distal femur (F2)

**Fig. 2:**
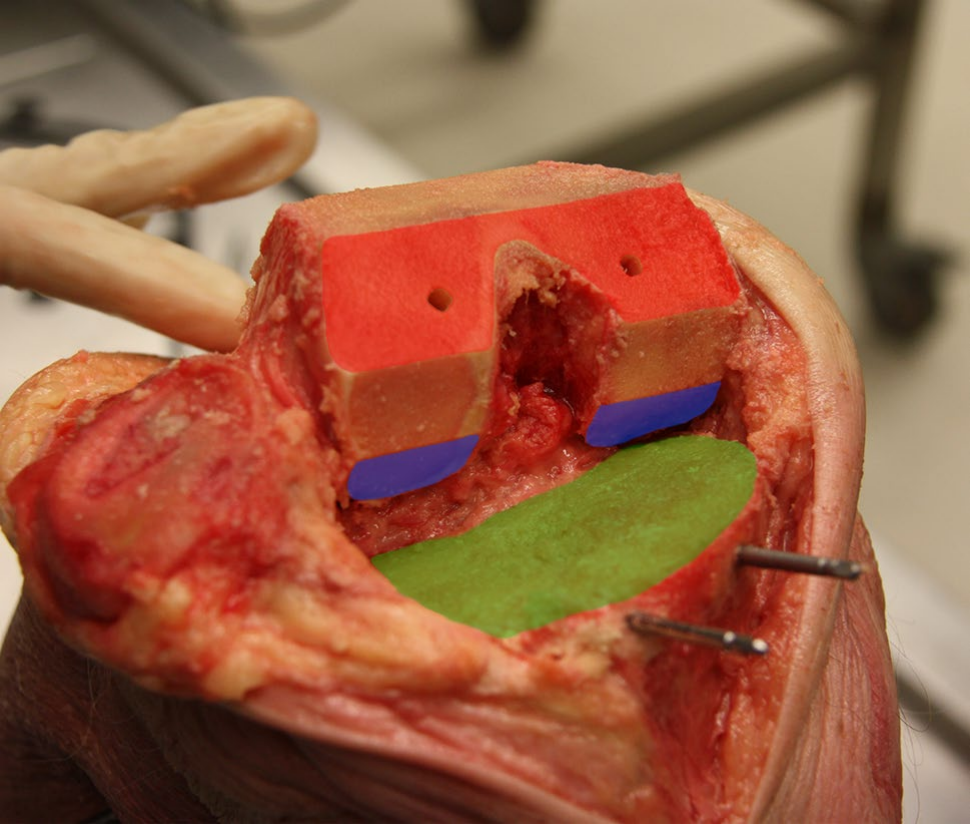
End result after all osteotomies. The distal femur (red), posterior condyle plane (blue) and proximal tibia plane (green) used for evaluation are shown

A single surgeon, with extensive experience in TKA surgery, carried out the surgical procedures. A standard medial parapatellar approach was used to expose the femur and tibia. Firstly, the tailor-made femur guide was positioned correctly in relation to the supplied 3D bone model. Once the surgeon was satisfied with the guide placement, the guide was fixed in place with four pins (Fig. 1). Subsequently, the cutting guide for the surgical saw was slid over the pins and the osteotomies were performed. Three planes were used for evaluation, i.e. the distal femur cut, the posterior femur cut and the proximal tibia cut (Fig. 2).

To ensure that the methods and surgery were comparable and executed as intended, all osteotomies were planned and performed in the same hospital using the *Signature* software.

### Comparing planned osteotomy planes with actual planes

**Fig. 3:**
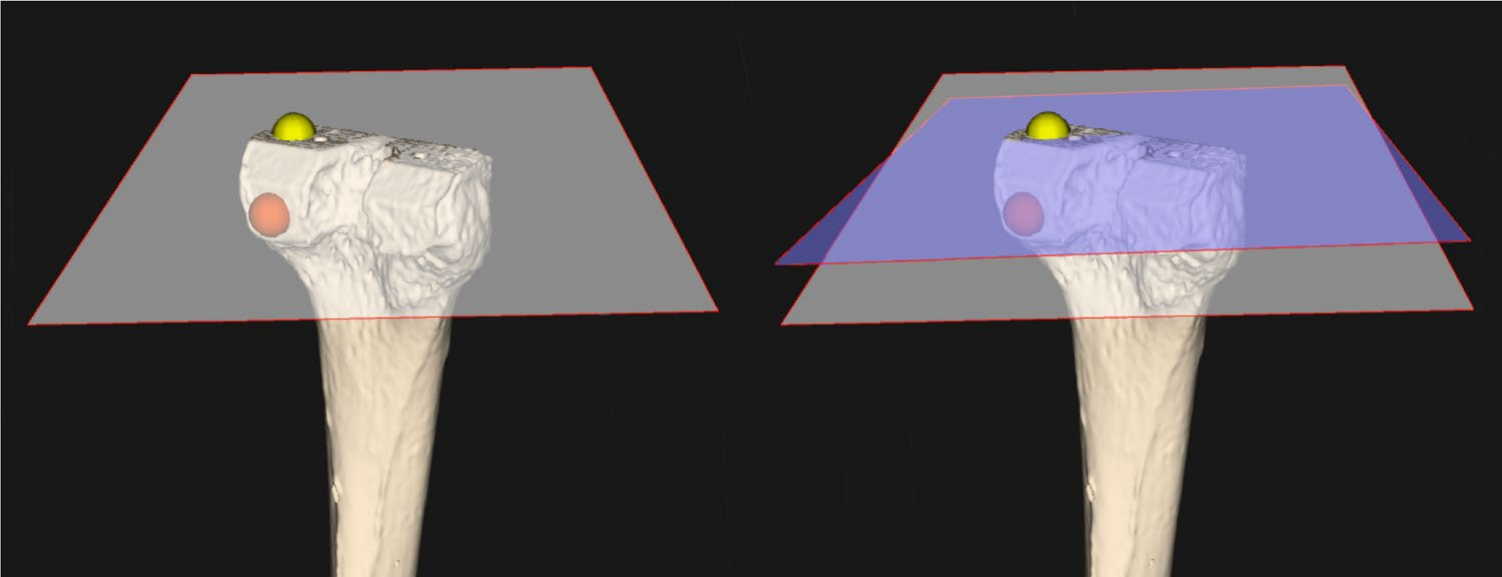
(Left) 3D spherical regions (only one region is shown per osteotomy) (red: posterior femur; yellow: distal femur) chosen for selecting points in the bone model for evaluating the cutting plane orientation. The plane fitted to the osteotomy is shown for the distal femur. (Right) The fitted plane (grey) deviates from the planned plane (blue)

**Fig. 4:**
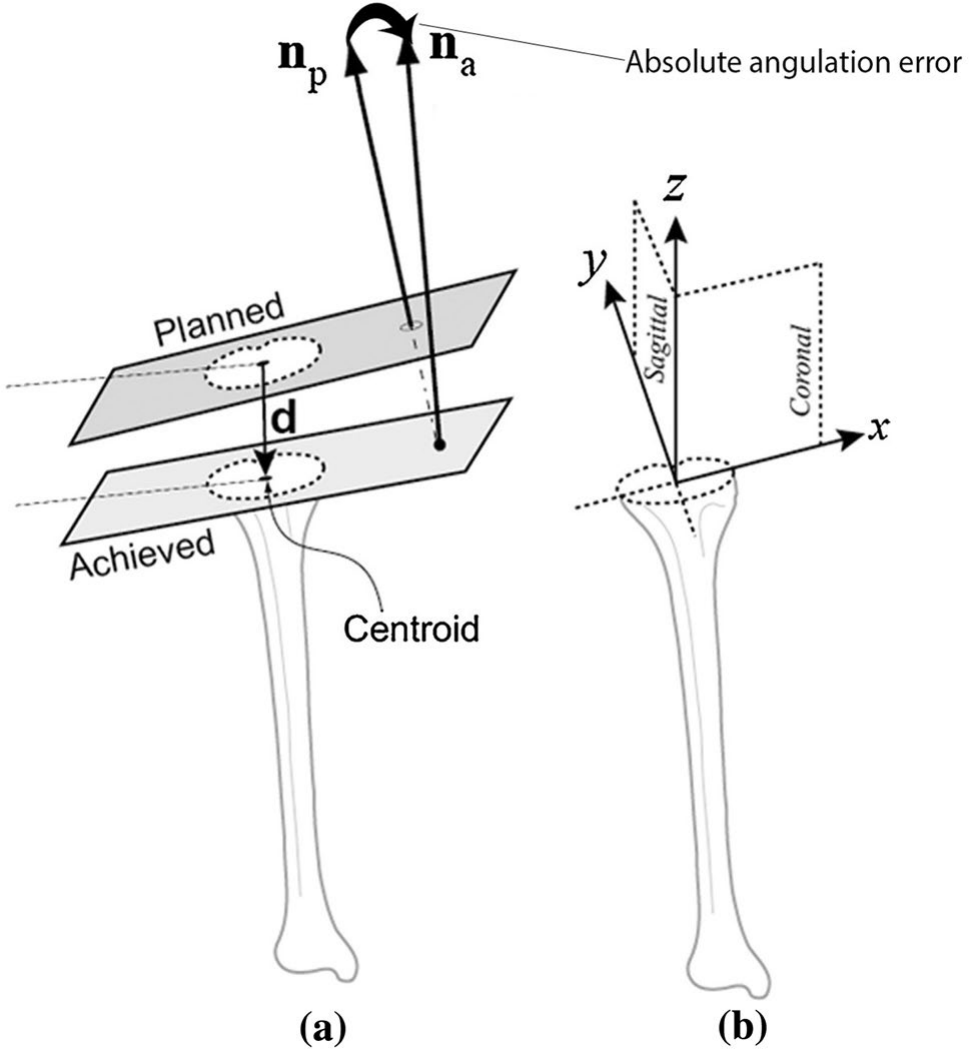
**a** Both the tibial planned as well as achieved planes with their corresponding normal vectors (*n*_planned_ = *n*_p_ and *n*_achieved_ = *n*_a_) and the absolute angulation error between them. **b** For a better clinical understanding of the difference, these vectors were also projected into the sagittal, coronal and axial planes to evaluate the angular errors in flexion and extension (sagittal) as well as varus and valgus (coronal) and rotation (axial)

To evaluate the postoperative osteotomies, another CT scan was made after the osteotomies had been performed. A validated and accurate method was used for assessment of the orientation and positioning of osteotomy cuts [10, 11]. This method uses the preoperative planning CT scan as a reference. The accuracy and reproducibility (test–retest was performed) of the method were below 0.2 mm for translations and 0.3° for rotations in the previous technical note. Therefore, differences between the planned and achieved osteotomy, which exceed methodological error, are believed to be caused by transfer errors. Following surgery, a further evaluation CT scan was made. The postoperative CT scan was used to create 3D polygons, digital models of the tibia and femur. After transforming the femur and tibia polygons into reference images, regions were selected to represent each polygon’s cutting plane. A position and a normal vector defined each plane. Several regions on the plane were sampled by automated selection of multiple points within a 3D sphere, positioned within the software (Fig. 3a). The corresponding plane that best fitted the average of these regions was determined and compared in terms of distance and rotational errors to the preoperatively predicted plane (Fig. 3b). Differences in the planned and achieved plane are expressed by the absolute angulation error and the distance error (Fig. 4a). The absolute angulation error is defined as the angle between the normal vectors of the planned and the achieved plane in 3D space. For a better clinical understanding of the difference, these vectors were also projected into the sagittal, coronal and axial planes to evaluate the angular errors in flexion and extension as well as varus and valgus and rotation (Fig. 4b). To this end, anatomical coordinate systems were defined for the femur and the tibia. An extensive explanation of this method and how the coordinate system was defined can be found in Dobbe et al. [11].

## Results

**Fig. 5:**
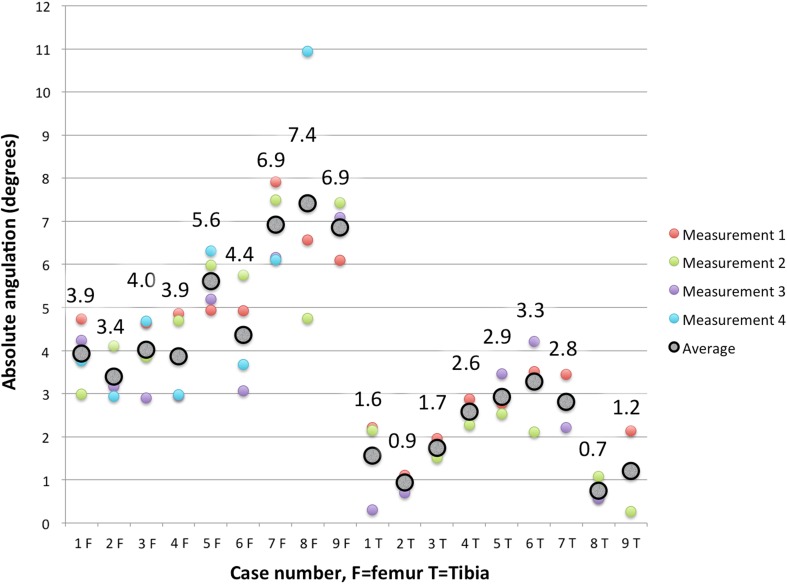
Absolute angulation difference, combined difference between planned and achieved planes for femur (1F-9F) and tibia (1T-9T). Black circles represent the average differences and colours are the different measurements per knee per osteotomy

For planes on the tibias, the average displacement error, d_err_ (± SD), of the system was 0.8 mm (± 0.5 mm). There was an absolute rotational error of 2.0° (± 0.9°) when compared to the predicted planes (Fig. 5). Broken down into coronal and sagittal projections, the rotational errors were 0.1° (± 1.2°) of varus and 0.4° (± 1.7°) of anterior slope (extension) (Table 1). For the femur, the average displacement error was 0.7 mm (± 4.0 mm). There was an absolute rotational error of 5.2° (± 1.6°) when compared to the planned planes (Fig. 5). Broken down into an average angulation difference of 2.8° (± 2.0°) in varus and 0.9° (± 2.7°) of flexion (Table 2). The average rotation about the *Z* axis of the femur was 1.4° (± 2.2°) of external rotation (Table 2). In general, transfer errors were smaller for planes on the tibia than the femur (Tables 1, 2, Fig. 5).

**Table 1:** Separate values of all three local tibial measurements with distance in mm and angulation errors in °

	Tibia		
	Distance along *Z* axis (mm) (*)	Varus (+)/valgus (−) (°)	Flexion (+)/extension (−) (°)
Case 1	−0.8	−0.9	1.0
Case 2	−1.3	0.2	0.7
Case 3	−0.6	0.5	−1.5
Case 4	−1.5	−1.2	2.1
Case 5	−1.5	2.6	−2.3
Case 6	0.1	0.9	−3.1
Case 7	−0.9	−0.8	−0.3
Case 8	−0.5	0.6	0.4
Case 9	−0.4	−0.8	−0.5
Average	−0.8	0.1	−0.4
SD of cases	0.5	1.2	1.7

The bottom row represents the standard deviation of the error parameters

*Negative values indicate that more bone was removed than intended

**Table 2:** Separate values of all four local femoral measurements with distance in mm and angulation errors in (°)

	Femur			
	Distance along *Z* axis (mm) (*)	Varus (+)/valgus (−) in (°)	Flexion (−)/extension (+) in (°)	External (+)/internal (−) rotation in (°)
Case 1	0.6	0.3	3.9	1.5
Case 2	−1.6	−0.6	−3.0	5.0
Case 3	−1.9	2.8	−2.8	1.2
Case 4	0.9	3.6	1.1	−1.4
Case 5	0.3	5.7	0.1	1.2
Case 6	10.9	3.4	−2.8	4.1
Case 7	−1.5	4.8	−5.0	1.4
Case 8	0.6	2.1	−3.4	0.7
Case 9	−1.8	3.4	1.9	−1.4
Average	0.7	2.8	−0.9	1.4
SD of cases	4.0	2.0	2.7	2.2

The bottom row represents the standard deviation of the error parameters

*Negative values indicate that more bone was removed than intended

## Discussion

The most important finding of this study is that the discrepancies for the tibia are so small that they can be considered to be clinically irrelevant. The discrepancies for planes on the femur were larger than those on the tibia. For the femurs, we saw systematic discrepancies in the plane orientation towards varus, flexion and external rotation. A combined (absolute error) of 5.2° is still impressive from a surgical perspective. The varus error, in particular, could change the mechanical axis and, therefore, the placement of the prosthesis. Too little slope might result in a slightly narrow flexion gap. This could be clinically relevant, as this would change the mechanical axis of the leg during walking. The flexion would be less of a problem because the curved shape of the prosthesis. A slight exorotation is preferable as it eases patellar tracking. However, rotational errors are also known to cause clinical complaints so this relevant for some patients. In one specimen (#6), there was a large error (> 1 cm), with too little bone osteotomized from the distal femur. It is not clear why this difference occurred but could be caused by incorrect placement of the cutting guide or osteotomy block, although the angular errors were not equally large in this specimen. In a live patient, this problem would be apparent and dealt with during surgery as placing the implant would be difficult because the extension gap would be too narrow. It would cause noticeable displacement of the joint line and a larger flexion gap if corrected by decreasing the implant size. Two specimens (#5 & #7) showed a varus error of around 5° which could produce a clinically relevant change of the mechanical axis of the leg and increased stress on the medial compartment. If the tibial osteotomy also caused too much varus (as in specimen #5, i.e., 2.6° of varus) the problem would be exacerbated.

Sawing with the oscillating saw from medial towards the lateral condyle could, in theory, explain the varus orientation. Under the assumption that cutting blade deflection increases with the distance from the cutting guide, errors are likely to be largest near the lateral condyle. Furthermore, after the saw blade passes through the medial condyle, it then bridges the intercondylar notch after which it will enter the lateral condyle at a slight angle because of anatomy. This may result in increased deflection of the saw compared to a situation where it enters at a 90-° angle, as for the tibia. However, after studying the data of our study in detail, it was clear that the most medial sections of the osteotomies show an average varus of 2.5° and the most lateral sections 2.5°. Therefore, it is unlikely that saw blade deflection is causal to the errors found in this study as the difference between medial and lateral is negligible. It is more likely that asymmetrical positioning of the femur guides would have caused the varus, flexion and external rotation. If the cutting-guide contact points with the femur are slightly higher distally than proximally, for instance because of remaining or overlapping cartilage interposition in the notch, this could result in more varus, flexion and external rotation than desirable. Another explanation could be that producing the guide is easier for the more proximal rounded part of the femur than, for instance, in the notch, which is anatomically more difficult to map.

Most of the previous reports on the accuracy of PSI systems use final implant position as the measure to judge positioning accuracy [6, 8, 15, 17, 18, 23]. Nam et al. [21] compared 41 knees implanted using CAS with 41 knees implanted using the *Signature* MRI-based PSI method. They noted that in the *Signature* PSI group, 88% of tibial components had an alignment within 2° perpendicular to the neutral mechanical axis. For the femoral components, 90% had an alignment within 2° perpendicular to the femur mechanical axis. Their results are better than the results reported in our study, but the numbers are difficult to compare, as they did not evaluate the osteotomies. In their study, the prosthesis orientation was measured by hand on plane radiographs, and no measurement error was given for the evaluation method. In a study by Ng et al. [22], 569 implants using *Signature* were reviewed retrospectively. Again the position of the implant was evaluated using long-leg radiographs. It was reported that the mechanical axis passed through the central third of the knee more often with *Signature* PSI (88%) than with manual instrumentation (78%). Furthermore, they reported that PSI had 10% outliers (> 2°) for the tibial component and 22% for the femoral component. The finding that the femoral orientation is less accurate is consistent with our study. A third study used postoperative CT to evaluate the *Signature* system in 23 TKA patients [24], but they only reported femoral implant rotation about the long axis. This study also used final implant position as a measure of success and also omits reporting the measurement error of the evaluation method and the variability in their observations. They did see median postoperative rotation of 0° for the femur as planned.

Some previous studies have tried to assess the transfer accuracy of other PSI systems more accurately by using computer navigation to assess the position and orientation of the cutting guide [7, 20]. Conteduca et al. reported that for 12 procedures the mean deviation of the tibial guide from the ideal alignment on the coronal plane was 1.2° (± 1.5°) and in the sagittal plane 3.8° (± 2.4°) [7]. On the coronal plane, the mean deviation of the femoral guide from the ideal alignment was 1.2° (± 0.6°) and in the sagittal plane was 3.7° (± 2°). Lustig et al. reported that for 60 procedures, the mean deviation of the tibial guide from the ideal alignment on the coronal plane was 0.6° (± 1.9°) and in the sagittal plane −0.1° (± 2.6°) [20]. On the coronal plane, the mean deviation of the femoral guide from the ideal alignment was 0.2° (± 1.8°) and in the sagittal plane was 2.1° (± 2.8°). No researchers investigated the system studied in this paper. The results described in these studies seem to corroborate the data reported in our study. However, computer navigation has been known to result in displacement errors of up to 2 mm caused by the effect of the distance between the stereoscopic camera system and surgical tools [27]. Furthermore, the computer navigation approach does not take into account that the achieved osteotomy might not have a direct relationship with the guide because of the saw blade deflection mentioned previously or changes made on basis of clinical judgment after placing the guides. Changes might also occur with the removal of the pin-positioning guides and placement of the saw guide. Therefore, the position of the guide might not actually be related to achieving the actual planned plane. Thus, the above-mentioned studies are not equipped to evaluate the transfer accuracy of the osteotomy plane itself. It could be hypothesized that cutting block guides produced with a slit to guide the saw directly are more accurate than pin-positioned guides because the first technique avoids an extra intraoperative step. One study reported on the mean discrepancies of distal femoral and proximal tibial cuts using the Visionaire systems, a cutting block-guided system, by measuring the thickness of the removed bone segment and comparing it to the planned values [28]. The mean discrepancy was reported to be 3.1 ± 1.0 and 3.1 ± 1.1 mm for distal femoral medial end lateral cuts, respectively, and 2.7 ± 0.9 mm for both proximal tibial medial and lateral cuts. In our study, an average discrepancy of 0.7 ± 4.0 mm for femoral cuts and 0.8 ± 0.5 mm for tibial cuts was found. Therefore, it seems that using a cutting block guide does not result in fewer discrepancies. However, it is arguable that the two measuring techniques are too diverse to make this comparison.

The accuracy and reproducibility of the method used in our study were below 0.2 mm for translations and 0.3° for rotations in the previous technical note [11]. Therefore, differences between planned and achieved osteotomies exceeding the methodological error are expected to be caused by transfer errors.

In the here reported study, CT scan data was used to plan and evaluate the osteotomy planes because CT scans yield high bone-soft tissue contrast which makes it easier to assess the transfer accuracy of the osteotomy planes. Therefore, our results cannot be applied to the MRI version of the *Signature* system. Further studies should systematically compare both CT and MRI systems to confirm which yields more accurate results. The downside of a guide produced on the basis of CT data is that it needs supporting points that lie outside the cartilage layer. MRI-produced guides sit adjacent to the cartilage layer and have a larger surface area to ensure adequate placement. However, MRI-produced guides have been shown to be less accurate than CT-produced guides in other systems [26].

There are some limitations to this study. The system currently studied is a pin-positioning guide. After placements of the pins, the guide needs to be replaced by a standard cutting block. This potentially introduces the risk of pin movement and thus decreased accuracy. Guides with a slotted saw blade sleeve could potentially be more accurate. Furthermore, this is a cadaveric study so the clinical effects and outcome cannot be measured and the results may not be transferrable to real-life total knee replacement surgery. Not all the cadavers had arthritic knees, so the positioning of the guides might be less accurate on osteophytic bones in a live patient. To position the guide for correct cutting, any soft tissue trapped between bone and guide could alter the orientation of the guide. In cadaver limbs, any obstructing soft tissues can simply be cut away and the guide positioned on the bone accurately. However, for patients, it is important to cause as little soft tissue damage as possible during surgery as this can impair the recovery process. Nevertheless, great care was taken to perform the operations as if on a live patient. It would have been preferable to analyse a larger number of specimens but the cost aspect limited us to a restricted number of specimens. However, the size of the study group is, in part, compensated by the highly accurate evaluation technique. Cost also prevented us from actually placing expensive implants so prosthesis positioning could not be evaluated. This has been studied by several other authors and was not the main focus of the here reported study. Finally, we only evaluate the most commonly used system (*Signature*) so the validity of this study for other systems is not necessarily transferrable.

## Conclusion

The production of guides produced by means of a 3D system based on CT data was assessed on cadaver specimen knees. The predicted osteotomy planes were more accurate for the tibia than for the femur. The use of 3D system techniques in TKA surgery provides accurate intraoperative guidance tailored to individual patients ensuring better placement of the implant, even for patients with bone deformities. Future studies could investigate further benefits such as reduced operation time, potentially fewer complications and longer implant survival with this method of controlled and improved component alignment.
